# BIOGAS: A Bright Idea for Africa

**DOI:** 10.1289/ehp.114-a300

**Published:** 2006-05

**Authors:** Valerie J. Brown

Ibadan, the second largest city in Nigeria, is the center of a large agricultural region in Oyo State. Since the nineteenth century, fierce intertribal rivalries and other political unrest have pushed large influxes of refugee and military populations into the city. This chaotic growth has discouraged the kind of municipal infrastructure that is taken for granted in the developed world. Soon, however, Ibadan’s power needs, at least, will get a boost from a relatively simple but extremely effective source of energy that is increasingly finding favor across Africa: biogas.

Biogas technology, which converts biological waste into energy, is considered by many experts to be an excellent tool for improving life, livelihoods, and health in the developing world. Worldwide, about 16 million households use small-scale biogas digesters, according to *Renewables 2005: Global Status Report*, a study by the Worldwatch Institute. The Ibadan plant will be one of the larger biogas installations in Africa, providing gas to 5,400 families a month at around a quarter the cost of liquefied natural gas.

The Ibadan digester will take advantage of the city’s Bodija Municipal Abattoir, where nearly two-thirds of the animals in Oyo State are slaughtered, according to a study in the January 2002 *African Journal of Environmental Assessment and Management*. The wastes from the slaughtering process are rinsed into open drains that connect to surface water; they also percolate into groundwater. About 60% of Ibadanians get water from hand-dug wells vulnerable to contamination from surface sources, and about 15% have private wells tapping a deep aquifer, according to Tijani Moshood, a geologist at the University of Ibadan.

Abattoir waste carries high levels of microorganisms that cause disease in humans and animals, such as *Salmonella* and *Escherichia coli* bacteria, Rift Valley fever virus, and parasites that cause toxoplasmosis and trichinellosis. Pesticides, antibiotics, metals, industrial chemicals, and the agents responsible for bovine spongi-form encephalopathy (BSE) may also enter the human food chain at an abattoir if they are present in the animals. Furthermore, decomposing organic material releases methane and carbon dioxide (CO_2_). CO_2_ is a primary culprit in climate change, but methane is even worse—23 times more potent than CO_2_, according to the Intergovernmental Panel on Climate Change report *Climate Change 2001: The Scientific Basis*.

Fortunately for the people of Ibadan, the new plant should mitigate many of these hazards. The project, dubbed Cows-to-Kilowatts, is a joint venture among the Nigerian branch of the Global Network for Environment and Economic Development Research, a nongovernmental organization (NGO); the Biogas Technology Research Centre of King Mongkut’s University of Technology in Thonburi, Thailand; the Centre for Youth, Family and the Law, a Nigerian NGO; and the Sustainable Ibadan Project, which is part of UN-HABITAT. Cows-to-Kilowatts was a 2005 winner of the Supporting Entrepreneurs for Environment & Development (SEED) Awards, which honor outstanding new entrepreneurial ideas for sustainable development worldwide.

Joseph Adelegan, a civil engineer and project director for Cows-to-Kilowatts, estimates the project will cost around US$300,000. Startup funds have been procured, and construction of the new plant is expected to begin by July 2006. The Ibadan system will employ a sophisticated design known as an anaerobic fixed-film digester, in which the active microorganisms are attached to an inert medium. The fixed-film technique shortens the time it takes for complete digestion, which enables the digester to be more compact.

## Nuts and Bolts

Biogas is one of many biomass energy sources, which include anything that was once alive and that can generate energy (except for fossil fuels, which are not renewable). In addition to direct use of wood and charcoal, biomass energy sources include ethanol and biodiesel. But these forms require considerably more investment, advanced technology, and/or resources than basic biodigesters provide. Ethanol, for example, requires advanced technology, whereas biodiesel, although relatively easy to produce, requires the availability of plant oil. Biogas technology simply formalizes the natural decomposition process.

A biogas digester consists of one or more airtight reservoirs into which a suitable feedstock—cow dung, human waste, abattoir waste—is placed, either in batches or by continuous feed. Small-scale digesters for household use are commonly made of concrete, bricks, metal, fiberglass, or plastic. Larger commercial biogas digesters are made mainly of bricks, mortar, and steel.

Digestion is accomplished in two general stages. First, acidogenic bacteria turn biomass into volatile fatty acids and acetic acid. Then methanogenic bacteria metabolize these compounds into a combination of methane-rich gas and an odorless phosphorus- and nitrogen-laden slurry, which makes excellent fertilizer. Depending on temperature and moisture content, it takes about 6–25 days to fully process a batch, according to a fact sheet from WASTE, a development NGO based in the Netherlands. Simpler digesters may take longer.

The end product is about 60–70% methane and 20–30% CO_2_, with small amounts of hydrogen sulfide and other impurities. The gas can be connected to a household stove for cooking, to a light fixture with a gauze mantle for lighting, or to other appliances with simple natural gas plumbing; it burns like liquefied petroleum gas. Depending on the design and size, prices for small-scale biodigesters run from US$100 to US$1,700.

It takes 1–2 cows, 5–8 pigs, or 4 adult humans to supply adequate daily feedstock for a single-household biodigester, according to a UNDP–Global Environment Facility fact sheet. The daily input of dung and urine from a single cow produces 1–2 kilowatt-hours of electricity or 8–9 kilowatt-hours of heat. Over a year, this is just about enough to run a refrigerator. In most African applications, a household biogas installation provides sufficient energy for cooking and some lighting.

## The Environmental Health Payoff

Properly designed and used, a biogas digester mitigates a wide spectrum of environmental undesirables: it improves sanitation; it reduces greenhouse gas emissions; it reduces demand for wood and charcoal for cooking, and therefore helps preserve forested areas and natural vegetation; and it provides a high-quality organic fertilizer. A well-maintained digester can last over 20 years and will pay for itself in one-fifth that time. But for the developing world, biogas’s greatest benefit may be that it can help alleviate a very serious health problem: poor indoor air quality.

Some 2 billion people around the world, including 89% of the sub-Saharan African population, use biomass for cooking and heating, according to *Energy for Development: The Potential Role of Renewable Energy in Meeting the Millennium Development Goals*, a report stemming from a 2004 conference of the same name organized by the Dutch government. Where combustible biomass is the chief energy source, life often revolves around an indoor cookstove or open fire that likely has no vent to the outdoors. Just gathering the fuel takes several hours a day—work that, in sub-Saharan Africa, is done almost entirely by women and children, according to *Energy for Development*. Since women also do most of the housework, including cooking, they and their children are exposed to cookstove smoke far more than men.

Their respiratory health suffers accordingly. In 2000, burning solid fuels caused 1–2 million deaths, comprising 3–4% of total global mortality, according to *Renewables 2005*. Indoor air pollution such as that stemming from biomass burning may increase the risk of acute lower respiratory infections in children, chronic obstructive pulmonary disease in adults, tuberculosis, low birth weight, asthma, ear infections, and even cataracts, according to the 2002 WHO report *Addressing the Links between Indoor Air Pollution, Household Energy and Human Health*. The Global Health Council, an international group of health care professionals and organizations based in Washington, DC, states that of all infectious diseases worldwide, those in the lower respiratory tract are the leading cause of death.

Clearly, biogas—being free of smoke—offers dramatic improvement of this particular health problem. Even so, concerns among potential users about other health risks of biogas generation have impeded more widespread adoption of the technology.

## The Question of Sanitation

A biogas digester can function well on human and animal waste. A quantity of liquid also is necessary; usually water is used, but urine works, too. Different kinds of waste can be mixed, although the cellulose and lignins in plant waste resist decomposition and may cause problems in the digester.

Some potential users are thus reluctant to try the digesters out of concern about sanitation, according to Dhananjay Kunte, a researcher in the Department of Internal Medicine at Evanston Northwestern Healthcare in Illinois, who has conducted several biogas pathogen reduction experiments funded by the government of India. In the developing world, this is no small worry. According to the Global Health Council, almost 40% of deaths in Africa are due to diarrheal diseases; the figure is even higher in Southeast Asia.

There is no question that human and animal waste is loaded with pathogens—*Salmonella*, *E. coli* O157:H7, *Campylobacter jejuni*, *Yersinia enterocolitica*, *Giardia lamblia*, and several types of *Cryptosporidium*, among others. Most of these pathogens are transmitted via the oral–fecal route and can cause diarrhea, abdominal cramps, dehydration, fever, vomiting, and—in vulnerable populations such as infants, children, the elderly, and immunocompromised persons—death. Even though the biodigestion process naturally reduces the pathogen load, handling biogas feedstock and using biogas slurry as fertilizer does carry some risk of infection.

It is not entirely clear whether digester slurry can still harbor enough pathogens to infect humans who handle it or eat crops fertilized with it. In several experiments using human waste as a feedstock, Kunte studied *Salmonella*, *Shigella*, and *Vibrio cholerae*, pathogens common in India that produce symptoms similar to those cited above. Kunte found that separating the overall digestion process into discrete acidifying and methanogenic stages—thereby isolating the acidogenic bacteria in their own tank—resulted in complete eradication of live pathogens. (Biodigesters probably can not break down the prions that cause BSE, although this is not known to have been tested. However, the risk of BSE is probably low in Africa because most cattle there are free-ranging and not fed cattle parts.)

Greg Austin, director of AGAMA Energy, a Cape Town, South Africa–based alternative energy company, says that once people see a digester in action and are trained in proper hygiene, such as washing their hands while working with it, they realize that health risks associated with operating a biodigester are relatively minor. Austin himself has installed a number of biogas systems in rural areas.

## Attitudes and Applications

Beyond concerns about sanitation, successful adoption of biogas in the developing world is highly dependent on political, economic, logistical, and social factors. Again, a key to successful adoption of biogas technology appears to be direct observation and experience. “The problem for anaerobic digester technology is that it is seen as complicated, but it really can be very simple,” says Paul Harris, an agricultural engineer at the University of Adelaide in Australia. “And because it is seen as complicated, it is regarded as hard and expensive, but many thousands of rural units worldwide show that this is not true.”

In 1982 Tanzania started distributing concrete-and-steel digesters that cost about US$1,400; by 1991 there were only 200 functioning biogas units in the country, according to an article by Innocent Rutamu in the July 1999 issue of *Livestock Research for Rural Development*. Rutamu, a development officer with the Tanga Dairy Development Programme in Tanzania, was testing a plastic unit that cost only US$50. He surveyed 72 farmers in the Tanga region and found that about half had heard of producing biogas from cow dung, but none were already using a digester. Three-quarters thought digesters would be expensive, but most of them could easily pay half the estimated construction cost of $50. Nearly all looked forward to not having to gather wood in the rainy season and no longer risking injury from snakes and thorns during firewood collection. Rutamu’s team distributed and installed 46 of the plastic digesters in several villages. After the digesters had been running for five months, respondents said they were doing an average of five fewer hours of housework per day.

Somewhat larger-scale biogas plants also operate successfully in a number of African locations. Biodigesters in five of Rwanda’s largest jails provide more than half of the prison kitchens’ energy, according to a 30 June 2005 BBC report. And a 30 November 2005 article in the Rwandan newspaper *The New Times* states that the Institute for Scientific Research and Technology in Kigali plans to install some 1,500 biogas digesters by 2009 in the *imidugudu* settlements, villages where rural Rwandans were relocated after the genocidal wars of the mid-1990s.

Other regions, too, have seen a reasonable amount of adoption, says Harris. Nepal celebrated the construction of its ten-thousandth unit a few years ago, and there are thousands of polyethylene digesters operating in Vietnam, as well as a huge number of Chinese and Indian gobar gas units.

In regions where there is already a mature electrical grid, there is limited incentive to use simple biogas digesters because they are not easily scaled up to produce energy comparable to hydropower and coal. Likewise, large farms and dairy operations need appropriately scaled treatments for the mountains of dung and waste their animals and crops generate. In developed markets, energy companies are seeking to convert 100% of biomass to energy, says Mark Kendall, an energy specialist in the renewable resource division of the Oregon Department of Energy. Using biogas alone has an energy conversion efficiency (the proportion of energy produced to that consumed) of about 10% or less, according to *Solid Waste Conversion: A Review and Database of Current and Emerging Technologies*, a 2003 report by the University of California, Davis, Department of Biological and Agricultural Engineering. By comparison, nonrenewable natural gas has an energy conversion efficiency of 55%. Austin counters, however, that this figure depends on conversion technology and energy type (for example, thermal or electrical). When used in a combined heat and power configuration, he says biodigester efficiency can approach 88%.

Still, with its sulfur compounds and other impurities, biogas is too dirty to feed directly into natural gas systems driving motors or to be used as transport fuel in place of gasoline. And in many African countries, bottled liquefied petroleum gas is used rather than natural gas due to lack of both infrastructure and large markets to justify investment in piped gas supply systems. Biogas is not easily bottled and thus must be used near its sources.

## The Bright Side

Basic biogas technology is therefore probably limited to places like sub-Saharan Africa—but in those places, it can make a big difference. In those environments, says Austin, the cost per unit of energy over a digester’s 15- to 20-year life cycle is lower than both solar electrification and the cost of extending a conventional electrical grid.

There is plenty of scope for biogas technology to expand in Africa. An AGAMA Energy fact sheet estimates that in South Africa there are 400,000 households with two or more cows and no electricity that could make use of biogas digesters. The fact sheet further notes that 45% of schools in South Africa have no electricity, 66% have poor sanitation facilities, 27% have no clean water, and 12% have no sanitation at all. Biogas installations could help mitigate all of these problems.

According to *Renewables 2005*, global energy demand nearly doubled between 1971 and 2002. Whether developed or developing, nations are caught between a rising population generating massive amounts of waste and the impending arrival of hard limits to nonrenewable energy sources. The need for clean, renewable energy is especially acute in the developing world, where few efficiencies have been introduced. In this context, biogas technology is a very good solution to local energy needs, and provides significant benefits to human and ecosystem health. Further expansion of biogas solutions via relatively inexpensive policy initiatives and the development of new technology combinations offers one very bright spot in the diminishing constellation of energy choices, wherever in the world they must be made.

## Figures and Tables

**Figure:**
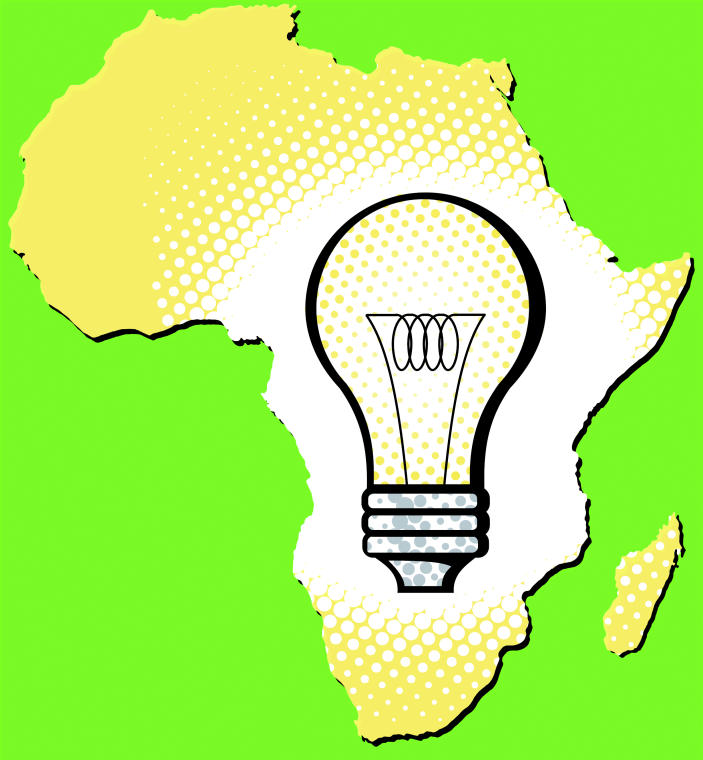


**Figure:**
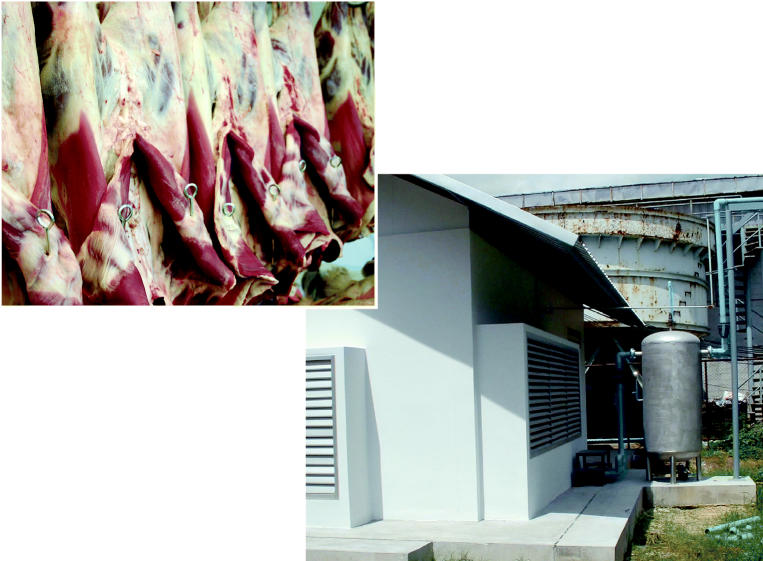
From abattoir to energy. A biodigester converts slaughterhouse waste into energy and solves two environmental problems—unhealthy waste and a need for power—at once.
